# Dynamic mechanical properties of different types of rocks under impact loading

**DOI:** 10.1038/s41598-023-46444-x

**Published:** 2023-11-06

**Authors:** Zixu Wang, Junhong Huang, Yanglong Chen, Xinping Li, Tingting Liu, Fei Meng

**Affiliations:** 1 School of Civil Engineering and Architecture, Wuhan University of Technology, Wuhan, 430070 China; 2 School of Resources and Environmental Engineering, Wuhan University of Technology, Wuhan, 430070 China; 3 Sanya Science and Education Innovation Park, Wuhan University of Technology, Sanya, 572024 China

**Keywords:** Engineering, Civil engineering

## Abstract

To study the mechanical properties of different types of rocks under impact loading, static mechanical parameter tests and split-Hopkinson pressure bar (SHPB) dynamic impact experiments were conducted on five typical rock specimens. The mechanical properties and failure modes of different rock specimens under the same static and dynamic loading were investigated. The differences between numerical simulation results and laboratory test results under different constitutive models in LS-DYNA were also compared and analyzed. The results show that with the increase of SHPB impact pressure (0.5–0.8 MPa), the stress peak values of granite, marble, and limestone also increase, while gypsum and reef limestone follow no particular trend. At the same time, both HJC and RHT constitutive models can simulate the laboratory impact test results of granite, marble, and limestone, however, the gypsum and reef limestone are not modelled by the HJC constitutive model, while the RHT constitutive model can describe the deformation-damage-failure process of rock specimens with different strengths. Therefore, the RHT model can better reflect the real deformation and failure of rocks.

## Introduction

In underground tunnel construction, rocky slope excavation, mining, and other engineering projects, the retained rock mass is subjected to disturbance from blasting loads to varying extents. A thorough understanding of the dynamic mechanical properties of rocks is crucial to the safety of engineering projects^1–4^. The load range with strain rates between 10^–1^ and 10^4^ s^−1^ is usually defined as the scope of rock dynamics research^5^.

The split-Hopkinson pressure bar (SHPB) experimental system is commonly used to study the dynamic mechanical properties of rocks under high strain rates^6–8^. Li et al.^9^ explored the dynamic mechanical properties and fracture characteristics of rock materials under impact loading through SHPB experiments. Shan et al.^10^ obtained the complete dynamic stress–strain curves of marble and granite using SHPB experiments. Su et al.^11^ used 3-d printing to prepare jointed rock specimens and explored the influences of impact strain rate on the dynamic mechanical properties of jointed rocks. Some researchers used an improved SHPB experimental system to study the dynamic mechanical behavior of rocks to good effect. Han et al.^12^ conducted static-dynamic coupled experiments on NSCB granite to explore the influences of axial preloading and loading rate on the dynamic mechanical properties of granite. Zhu et al.^13^ and Gong et al.^14^ studied the dynamic response characteristics of diorite and sandstone under different impact load regimes using an improved SHPB experimental system and determined the influences of different strain rates and confining pressures on the dynamic mechanical properties of rocks. Wei et al.^15^ and Zhao et al.^16^ studied the dynamic mechanical properties of conglomerate and sandstone and improved the efficacy of the SHPB experimental system in terms of the dynamic mechanical properties of rocks.

With the interdisciplinary development of computer technology, numerical simulation methods have become a powerful research tool in geotechnical engineering^17–19^. Wang et al.^20^ conducted SHPB impact test simulations on marble using dynamic finite element software and found that the dynamic compressive strength of rock specimens was proportional to the impact speed; when the impact speed remains constant, the more numerous the impacts, the greater the strain rate in the rock specimens. Chunyu et al.^21^ used two-dimensional particle flow software to study the effects of strain rate and temperature on frozen soil crack propagation under impact loading. Tu et al.^22^ analyzed the damage parameter values in the RHT constitutive model. Zhang et al.^23^ reproduced the SHPB impact test on the LS-DYNA3D platform and simulated the fracture characteristics of rock mass under explosive impact using the HJC constitutive model parameters obtained by inversion. Wang et al.^24–26^ compared the simulation effects of RHT and HJC constitutive models under cyclic blasting action and believed that the RHT constitutive model can better reflect the real deformation and failure process of such rock specimens. Deng et al.^27,28^ used numerical simulation software systems to estimate the influences of the distribution and mechanical properties of rock mass joints under dynamic loading on the stability of underground space.

In summary, some scholars have produced many results pertaining to the dynamic mechanical properties of rock^29–33^, but there are relatively few studies on the differences in dynamic mechanical properties between different types of rocks. In the present work, static mechanical parameter tests and dynamic mechanical property analyses were conducted on five typical rock specimens, obtaining the static and dynamic mechanical properties and failure modes of rock materials, and the applicability of HJC and RHT constitutive models in rock dynamics was compared and analyzed.

## Static mechanical tests on rock specimens

**Figure 1:**
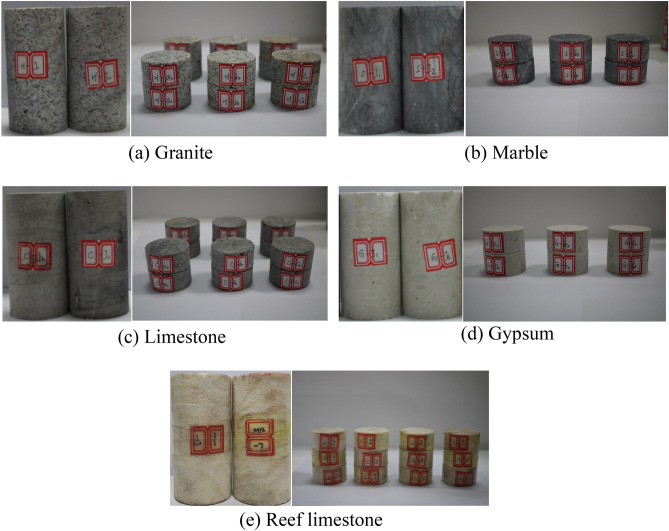
Different types of rock specimens.

In order to obtain the mechanical parameters required for the numerical model, this paper carried out both static and dynamic mechanical tests. The numerical model parameters were calibrated through static tests, which were then used to validate the results of the dynamic tests. To obtain the static properties of different types of rocks, this study selected five rock types, including granite, marble, limestone, gypsum, and reef limestone. Parameters, such as density, elastic modulus, Poisson’s ratio, strength, and acoustic wave velocity, were measured. The collected rock samples were prepared into multiple sets of cylindrical specimens with a diameter of 50 mm and a height-to-diameter ratio of 2:1, as well as disk specimens with a diameter of 50 mm and a height-to-diameter ratio of 1:2 for static mechanical testing. The processed specimens are shown in Fig. 1.

### Density measurement

Density measurements were conducted on rock specimens in dry and saturated conditions. First, the length and diameter of the rock specimens were measured using a digital Vernier caliper with a resolution of 0.01 mm to calculate the volume of the specimens. An electronic balance with an accuracy of 0.01 g was used to weight the specimens. The ratio of the mass to the volume of the specimens gives the density of the rock specimens.

### Uniaxial compressive strength test

**Figure 2:**
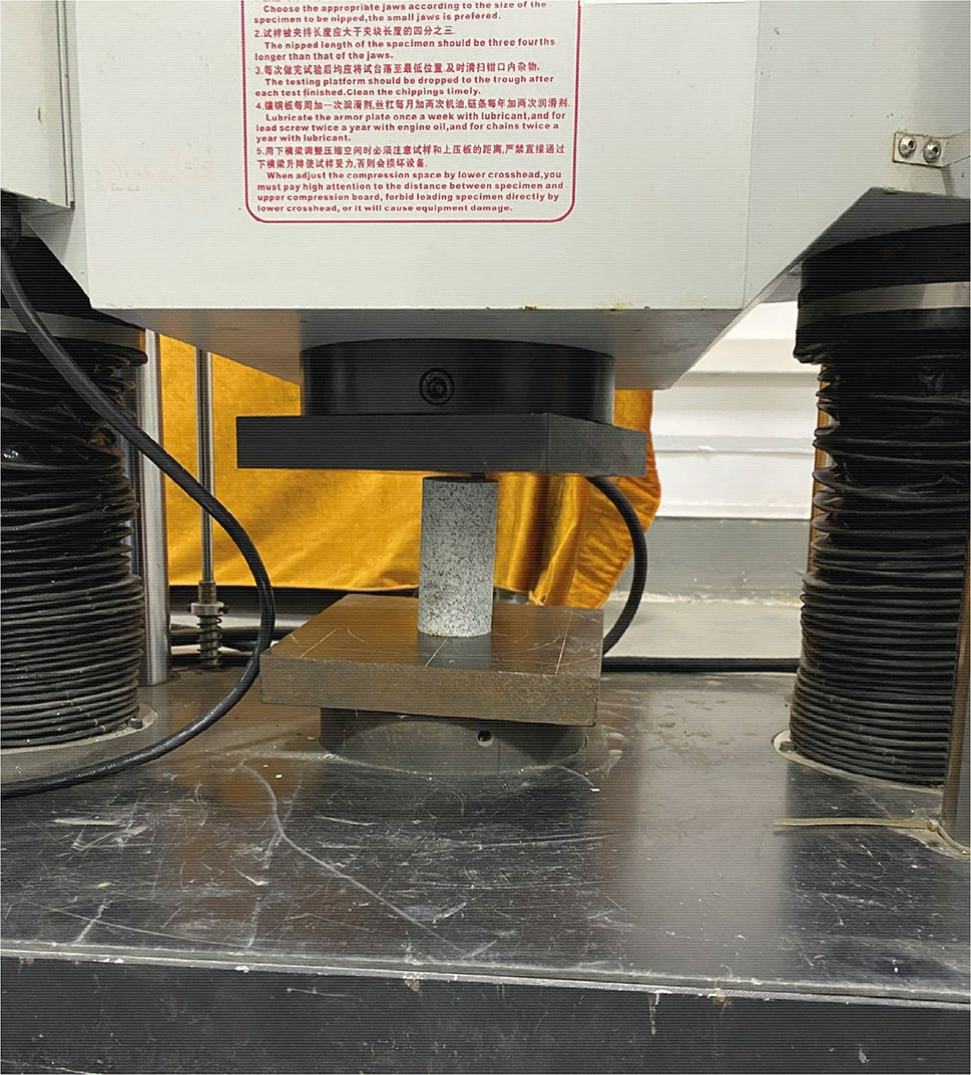
The uniaxial compression test process for rocks.

The uniaxial compressive strength of the rock specimens was tested using a SHT4106 microcomputer-controlled electro-hydraulic servo universal testing machine with a maximum load of 1000 kN, which consists of a load-application system and a strain-monitoring system. Figure 2 shows the process of the uniaxial compression test. Three specimens of each rock type were tested. The loading rate was set to 0.5 mm/min. The slope of the stress–strain curve in the elastic stage represents the elastic modulus. Meanwhile, the Poisson’s ratio of the specimens was determined by measuring the transverse and longitudinal deformation of the materials using strain gauges attached to the specimens.

### Brazilian splitting tensile strength test

**Figure 3:**
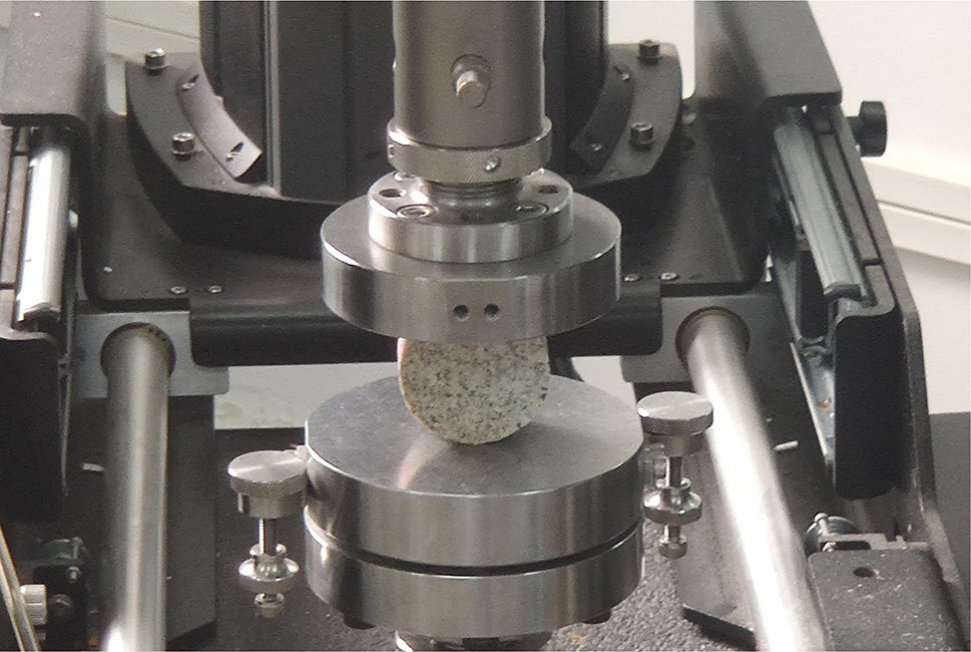
Rock splitting tests.

The Brazilian splitting method is recommended by the ISRM as the standard method for measuring the dynamic tensile strength of rocks^34^. Disk-splitting tests were performed on all types of rock specimens at a loading rate of 0.40 MPa/s. The loading conditions of some rock specimens are shown in Fig. 3.

### Acoustic wave velocity measurement

In recent years, methods based on elastic wave characteristics for testing rock properties have been widely applied in geotechnical engineering^35^. By measuring the P-wave velocity of rocks, the physico-mechanical properties of rocks can be indirectly understood. The RSM-SY5 (T) non-metallic acoustic wave detector was used to measure the P-wave velocity with a testing accuracy of 1 μs. The ultrasonic pulse method was adopted with a sampling interval of 0.1 μs, and a pressure of approximately 0.05 MPa was applied to the P-wave transducer.

Table 1 lists results from static mechanical tests on rock specimens: there are significant differences in the physico-mechanical properties of the five types of rock specimens. The elastic modulus ranges from 9.7 to 144 GPa, the uniaxial compressive strength ranges from 3.1 to 81.2 MPa, the tensile strength ranges from 1.11 to 7.79 MPa, and the average P-wave velocity ranges from 2694 to 6130 m/s. The differences in dry density, saturated density, and Poisson’s ratio are insignificant. It is worth noting that although the density of reef limestone is slightly lower than that of gypsum, its uniaxial compressive strength is much larger than that of gypsum. This is due to the unique skeletal structure of reef limestone, which gives it a certain strength, while the large number of pores therein reduce its density.

**Table 1:** Basic mechanical test data (average values).

Specimens	Dry density (g/cm^3^)	Saturated density (g/cm^3^)	Poisson's ratio	Elastic modulus (GPa)	Compressive strength (MPa)	Tensile strength (MPa)	P-wave velocity (m/s)
Granite	2.65	2.66	0.13	144.00	81.20	7.79	6130
Marble	2.70	2.70	0.17	121.00	55.80	5.67	5214
Limestone	2.76	2.79	0.20	116.00	28.50	5.06	4759
Gypsum	1.14	1.67	0.30	13.00	3.10	1.11	3338
Reef limestone	0.98	1.37	0.25	9.70	15.00	1.42	2694

## Dynamic impact tests on rock specimens

### SHPB test set-up

**Figure 4:**
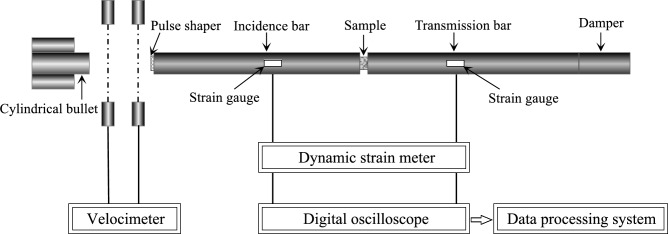
Equipment for SHPB tests.

The SHPB system was employed for impact tests, as illustrated in Fig. 4. The cylindrical bullet, incident bar, and transmission bar in the test system were all made of steel. The experiment used strain gauges affixed to the incident and transmission bars to measure the incident, reflected, and transmitted waves.

### Test preparation

Cylindrical specimens with good machining quality were selected for the SHPB impact test. A rubber disc was attached to the incident bar end as a pulse shaper to eliminate partial waveform dispersive oscillations and achieve a more regular experimental result curve. Based on preliminary impact test results, impact tests were designed with four different impact pressures: 0.5 MPa, 0.6 MPa, 0.7 MPa, and 0.8 MPa.

### Test equilibrium verification

**Figure 5:**
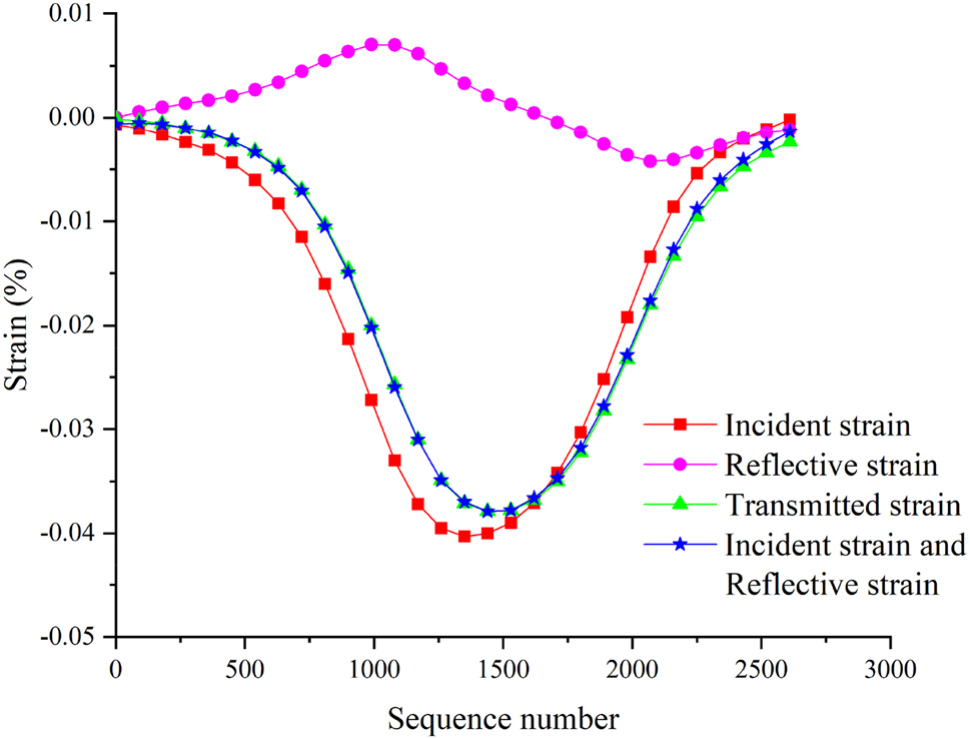
The stress equilibrium diagram of specimens.

Following the dynamic compression strength testing scheme suggested by ISRM^36^, the stress equilibrium diagram of rock specimens is shown in Fig. 5. The sum of the incident signal curve and the reflected signal curve coincides with the transmitted signal curve, indicating that the stress balance standard was achieved during the test, ensuring the reliability of the test results.

### Test results

The effectiveness of the SHPB test is based on two assumptions: stress uniformity and one-dimensional stress wave propagation. The purpose of stress uniformity was to decouple the inertia and rate effects of the specimen during the loading process and achieve quasi-static loading of the specimen. In the present work, the “three-wave method” was used to process data, and the stress $\sigma_{s}$, strain rate $\dot{\varepsilon }_{s}$, and strain $\varepsilon_{s}$ of the specimen were calculated through strain signals $\varepsilon_{in} (t)$, $\varepsilon_{re} (t)$, and $\varepsilon_{tr} (t)$ on the incident and transmitted bars, as given by Eq. (1):

1 $$\begin{aligned} \sigma_{s} & = \frac{{E_{bar} A_{bar} (\varepsilon_{in} + \varepsilon_{re} + \varepsilon_{tr} )}}{{2A_{spc} }} \\ \dot{\varepsilon }_{s} & = C\frac{{\varepsilon_{in} - \varepsilon_{re} - \varepsilon_{tr} }}{{L_{spc} }} \\ \varepsilon_{s} & = \frac{C}{{L_{spc} }}\int_{0}^{t} {(\varepsilon_{in} - \varepsilon_{re} - \varepsilon_{tr} )dt} \\ \end{aligned}$$

where:$A_{spc}$ is the cross-sectional area of the specimen, and $L_{spc}$ is the length of the specimen.

**Figure 6:**
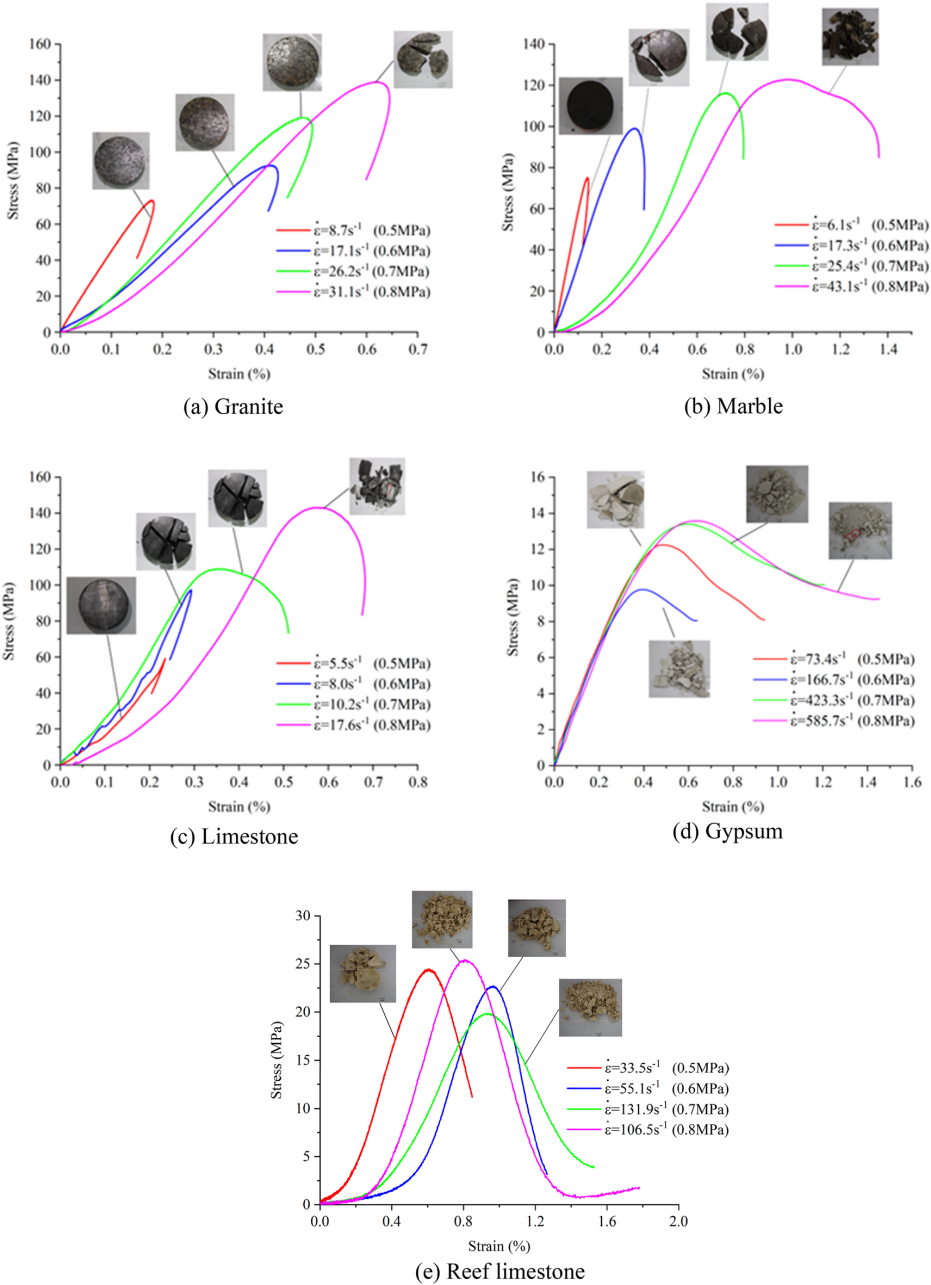
Stress–strain curves and failure modes of rock specimens at different strain rates.

The stress–strain curves and failure modes of the same kind of rock specimen under different impact loadings, i.e., different strain rates, are shown in Fig. 6. As illustrated in Fig. 6a–c, the strain rate of the three types of rocks increases with the increase in impact loading, and the area enclosed by the hysteresis loop in the stress–strain curve increases; however, the hysteresis loop of the granite stress–strain curve remains closed, while that of marble and limestone becomes open when the degree of fragmentation reaches a certain range, indicating that the strength of the rock specimens is very low. Under the same impact pressure, the peak stress intensity and degree of damage of limestone and marble are similar. Comparing the strain rates of the two, it is found that the strain rate of limestone is much smaller than that of marble under the same impact loading and fragmentation pattern. This finding indicates that the strain rate of the rock specimen is not only related to the degree of fragmentation and the size of the impact loading but also to the properties of the material.

Comparing the stress–strain curves of different rocks in Fig. 6, it can be observed that with the increase in impact load, the peak stress and peak strain of the rocks gradually increase. When the impact load is 0.8 MPa, the peak stress for granite, marble, limestone, gypsum, and reef limestone is 144.97 MPa, 122.9 MPa, 109.01 MPa, 13.72 MPa, and 25.46 MPa, respectively. Comparing limestone and marble materials, the two are close in strength, and the degree of damage under similar loading rates is also the same. However, when comparing strain rates at the same loading rates, it is observed that at low and high loading rates, the strain rates of both rocks are similar. Yet, at moderate loading rates, although the damage patterns are similar, there is a noticeable difference in strain rates, with marble's strain rate nearly twice that of limestone.

Reef limestone and gypsum belong to extremely soft rocks, showing evident damage under impact loads of 0.5–0.8 MPa. Gypsum has good homogeneity, and with increasing loading rates, the overall trend of peak strength is upward. However, at higher loading rates, the growth is minimal, and the damage state has turned into a powdery form. Reef limestone, with poor homogeneity among samples, does not exhibit a clear pattern in peak strength under the impact loads, indicating significant variability.

Gypsum and reef limestone belong to the category of extremely soft rocks, and their dynamic compressive strength peak value does not exceed 25 MPa. The dynamic elastic modulus of gypsum remains almost unchanged under different impact load regimes. Reef limestone, a porous material, has a longer compaction section and a larger discreteness in the stress–strain curve under impact load. As the impact loading increases, the degree of damage gradually increases, but the peak stress and strain rates do not show a significant trend. The main reason is the large discreteness of reef limestone, and its strength is greatly affected by reef-building corals, porosity, pore shape, and size.

## Numerical simulations

Numerical simulations were less affected by external environmental factors, and under certain conditions with similarity to experimental results, they can provide more universally applicable conclusions, making them suitable for widespread use in laboratory tests and practical engineering applications. For continuous damage constitutive models, both the HJC and RHT models can describe the mechanical properties of rock materials under high strain rates and large deformations well. However, in a single blast simulation, the damage cloud map of the HJC model can only characterize the formation of the pulverized zone and cannot describe the evolution process of radial cracks; while the RHT model can describe the formation of the pulverized zone near the blast hole and the extension of radial tensile cracks at the far end well^37–41^. Moreover, its simulation results are in good agreement with theoretical and experimental results.

### Establishment of the computational model

**Figure 7:**
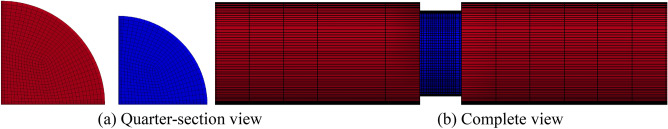
Grid division of bars and specimens in numerical simulation.

In order to obtain the mechanical parameters required for the numerical model, this paper carried out both static and dynamic mechanical tests. The numerical model parameters were calibrated through static tests, which were then used to validate the results of the dynamic tests^42,43^. Since the SHPB test has a symmetrical structure in the up-down and left–right directions, a quarter-symmetry model was chosen for numerical simulation using LS-DYNA to save computation time and disk space. Each edge could be evenly divided into 25 sections, and no constraints were applied to the bars in any direction. This allowed for an idealized SHPB test. The grid division of the bars and specimens in the numerical simulation is shown in Fig. 7.

The parameters used for the incident and transmitted bars were measured physical parameters, with density *ρ* = 7767 kg/m^3^, elastic modulus *E* = 205.6 GPa, and Poisson’s ratio *ν* = 0.2. The widely-used HJC constitutive model and RHT constitutive model were selected for the numerical modelling of rock specimens under test.

### HJC constitutive model

The HJC constitutive model could be used for brittle materials such as rock and concrete under large strain rates, high strain rates, and high pressures. The equivalent strength is expressed as a function of pressure, strain rate, and damage; pressure is a function of volumetric strain, including the effects of permanent fragmentation; and damage accumulation is a function of plastic volumetric strain, equivalent plastic strain, and pressure.

The expression for the HJC strength model is given by in Eq. (2):

2 $$\sigma^{ * } = \left[ {A\left( {1 - D} \right) + BP^{ * N} } \right]\left[ {1 + C\ln \left( {\dot{\varepsilon }^{ * } } \right)} \right] \le S_{\max }$$

where,$\sigma^{*}$ represents the normalized equivalent stress, $\sigma^{ * } = \sigma /f_{c}$, $\sigma$ represents the actual equivalent stress, $f_{c}$ represents the quasi-static uniaxial compressive strength of materials, $\dot{\varepsilon }^{*}$ refers to the normalized strain rate, $\dot{\varepsilon }^{*} = \dot{\varepsilon }/\dot{\varepsilon }_{0}$_,_ $\dot{\varepsilon }$ is the loading strain rates, $\dot{\varepsilon }_{0}$ is the reference strain rate (set to 1.0); $D$ denotes the damage, $P^{*}$ is the normalized pressure, $P^{*} = P/f_{c}$, $P$ is the hydrostatic pressures; $A$, $B$, $C$, and $N$ are the material model parameters, representing the normalized cohesive strength, normalized pressure-hardening, strain rate coefficient , and pressure hardening exponent, respectively; $S_{\max }$ is the maximum value of the normalized equivalent stress.

The HJC constitutive model describes damage based on the accumulation of equivalent plastic strain and plastic volumetric strain, with damage evolution equations as shown in Eqs. (3) and (4):

3 $$D = \sum {\frac{{\Delta \varepsilon_{P} + \Delta \mu_{P} }}{{\varepsilon_{P}^{f} + \mu_{P}^{f} }}}$$

4 $$\varepsilon_{P}^{f} + \mu_{P}^{f} = D_{1} \left( {P^{*} + T^{*} } \right)^{{D_{2} }}$$

where, $\Delta \varepsilon_{p}$ and $\Delta \mu_{p}$ represent the increments of equivalent strain and plastic volumetric strain within a single calculation cycle; $\varepsilon_{p}^{f}$ and $\mu_{p}^{f}$ stand for the equivalent plastic strain and plastic volumetric strain at failure under normal pressure; $T^{*} = T/f_{c}$ is the normalized tensile strength; $T$ is the material's maximum tensile strength; *D*_1_ and *D*_2_ are damage parameters for these materials.

Based on the experimental data and previous research results^24^, the final HJC model parameters were chosen as shown in Table 2.

**Table 2:** HJC constitutive model material parameters.

Variable	Granite	Marble	Limestone	Gypsum	Reef limestone
Mass density RO (kg/m^3^)	2760	2430	2120	1140	930
Shear modulus G (GPa)	63.7	51.7	48.3	5.0	3.9
Normalized cohesive strength A	0.57	0.57	0.57	0.57	0.57
Normalized pressure-hardening B	2.5	2.5	2.5	2.5	2.5
Strain rate coefficient C	0.0127	0.0127	0.0127	0.0127	0.0127
Pressure-hardening exponent N	0.79	0.79	0.79	0.79	0.79
Quasi-static uniaxial compressive strength FC (MPa)	81.2	55.8	28.5	3.1	1.42
Maximum tensile hydrostatic pressure T (MPa)	7.79	5.67	5.06	1.11	1.42
Amount of plastic strain before fracture EFMIN	0.01	0.01	0.01	0.01	0.01
Normalized maximum strength SFMAX	5	5	5	5	5
Crushing pressure PC (MPa)	27.1	18.6	9.5	1.0	5.0
Crushing volumetric strain UC	0.4	0.3	0.1	0.1	0.8
Locking pressure PL (GPa)	1.50	1.10	0.63	0.23	0.059
Locking volumetric strain UL	0.013	0.004	0.029	1.114	0.953
Damage constant D1	0.04	0.04	0.04	0.04	0.04
Damage constant D2	1	1	1	1	1
Pressure constant K1 (GPa)	12.0	12.0	12.0	12.0	12.0
Pressure constant K2 (GPa)	25.0	25.0	25.0	25.0	25.0
Pressure constant K3 (GPa)	42.0	42.0	42.0	42.0	42.0
Failure parameter FS	0.1	0.1	0.1	0.1	0.1

### RHT constitutive model

**Figure 8:**
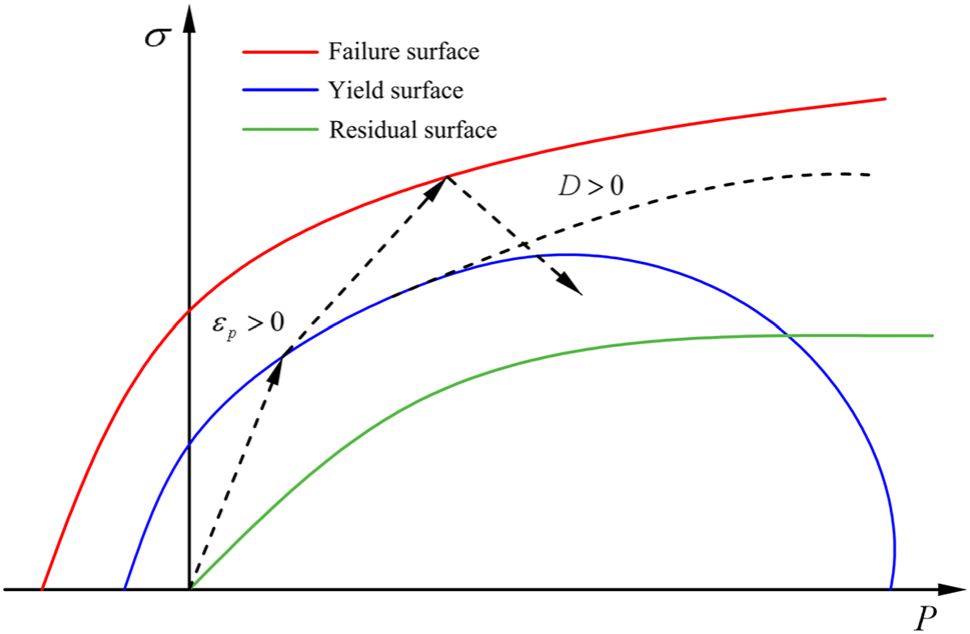
RHT model limit surfaces and loading scenarios.

The RHT constitutive model is a tension–compression damage model based on the HJC constitutive model and is an advanced damage-plasticity model suitable for brittle materials such as concrete and rock. It defines three limit surfaces to describe the changes in strength of the material, namely the yield surface, failure surface, and residual surface. The relationships between the three limit surfaces are shown in Fig. 8. In the RHT model, the damage variable $D$ is used to define the damage effect, which is the ratio of the accumulated equivalent plastic strain increment $\Delta \varepsilon_{p}$ to the final failure equivalent plastic strain $\varepsilon_{p}^{f}$, as shown in Eq. (5):

5 $$D = \sum \frac{{\Delta \varepsilon_{p} }}{{\varepsilon_{p}^{f} }}$$

When $D = 0$, it indicates no damage, and when $D = 1$, it denotes complete damage.

In numerical simulations, the choice of material model parameters has a decisive impact on the accuracy of the simulation results. Based on laboratory tests and previous research results^44,45^, the final RHT model material parameters were determined (Table 3).

**Table 3:** RHT constitutive model material parameters.

Variable	Granite	Marble	Limestone	Gypsum	Reef limestone
Mass density RO (kg/m^3^)	2760	2430	2120	1140	930
Elastic shear modulus (GPa)	63.7	51.7	48.3	5.0	3.9
Eroding plastic strain EPSF	2.0	2.0	2.0	2.0	2.0
Failure surface parameter A	2.5	2.5	2.5	2.5	2.5
Failure surface parameter N	0.85	0.73	0.71	0.70	0.70
Compressive strength FC (MPa)	81.2	55.8	28.5	3.1	50
Relative shear strength FS*	0.07	0.07	0.07	0.07	0.07
Relative tensile strength FT*	0.10	0.10	0.18	0.36	0.09
Lode angle dependence factor Q0	0.75	0.75	0.74	0.68	0.62
Lode angle dependence factor B	0.01	0.01	0.01	0.01	0.01
Parameter for polynomial EOS	0	0	0	0	0
Reference compressive strain rate EOC	3e−5	3e−5	3e−5	3e−5	3e−5
Reference tensile strain rate EOT	3e−6	3e−6	3e−6	3e−6	3e−6
Break compressive strain rate EC	3e−25	3e−25	3e−25	3e−25	3e−25
Break tensile strain rate ET	3e−25	3e−25	3e−25	3e−25	3e−25
Compressive yield surface parameter GC*	0.85	0.85	0.85	0.85	0.85
Tensile yield surface parameter GT*	0.4	0.4	0.4	0.4	0.4
Shear modulus reduction factor XI	0.25	0.25	0.25	0.25	0.25
Damage parameter D1	0.025	0.025	0.025	0.025	0.025
Damage parameter D2	1	1	1	1	1
Minimum damaged residual strain	0.01	0.01	0.01	0.01	0.01
Residual surface parameter AF	2.5	2.5	2.5	2.5	2.5
Residual surface parameter NF	0.85	0.85	0.85	0.85	0.85
Gruneisen gamma GAMMA	0	0	0	0	0
Hugoniot polynomial coefficient A1 (GPa)	43.87	43.87	43.87	43.87	43.87
Hugoniot polynomial coefficient A2 (GPa)	49.4	49.4	49.4	49.4	49.4
Hugoniot polynomial coefficient A3 (GPa)	11.62	11.62	11.62	11.62	11.62
Crush pressure (MPa)	133	110	90	70	75
Initial porosity	1.0	1.0	1.02	1.53	1.49

### Comparison and analysis of different constitutive models

#### Failure characteristics of rock specimens

**Figure 9:**
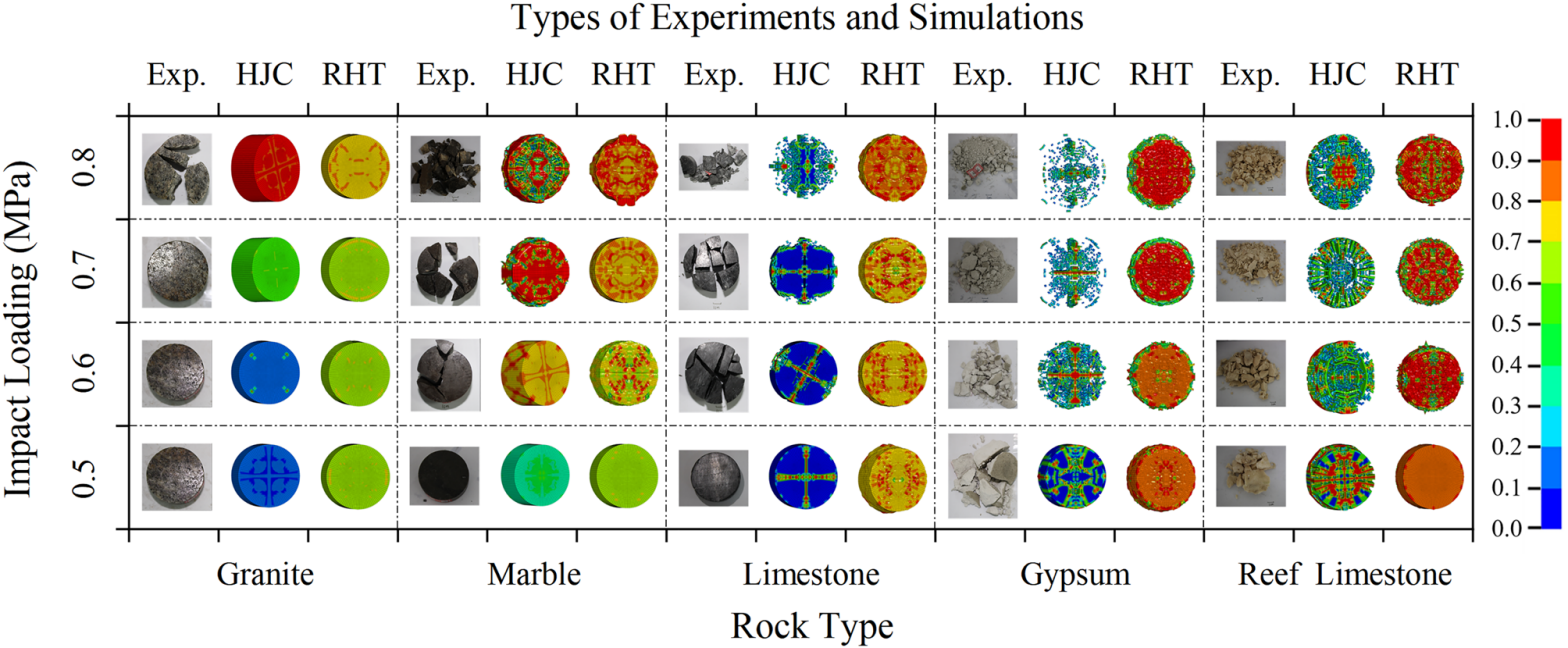
Comparisons of rock specimen failure process and damage cloud diagrams under different impact pressures.

The mechanical properties and failure modes of the five types of rock specimens were analyzed using the HJC and RHT constitutive models (Fig. 9). It can be found that both constitutive models return results with a certain similarity to the laboratory impact test results, indicating the feasibility and accuracy of the numerical simulation. When the impact pressure is 0.5 MPa, granite and marble show no significant deformation, limestone exhibits a certain degree of damage, while gypsum and reef limestone already show a trend of initial failure; when the impact pressure increases to 0.6 MPa, granite remains in the elastic stage, marble shows larger deformation, limestone exhibits severe damage, and gypsum and reef limestone present different failure modes; as the impact pressure is increased to 0.7 MPa, granite still shows no signs of failure, marble exhibits significant deformation, limestone undergoes local large-scale destruction, reef limestone experiences fragmentation failure, and the fragmentation failure mode of gypsum becomes more severe; finally, when the numerical simulation impact pressure is set to 0.8 MPa, granite shows signs of damage, the failure of the marble becomes more apparent, limestone undergoes large-scale failure, some rock blocks are fragmented, and gypsum and reef limestone show further fragmentation at failure.

Comparing the numerical simulation results of the HJC and RHT constitutive models, it can be found that when the strength of the rock specimen is relatively high, such as granite, marble, and limestone, both constitutive models can simulate the laboratory rock impact test results well; when the strength of the specimen is relatively low, such as in gypsum and reef limestone, rock specimens under the HJC constitutive model tend to fail when the deformation reaches a certain amount, and the model cannot fully represent the deformation of the rock specimens. The main reason is that the HJC constitutive model does not consider the third invariant of the deviatoric stress tensor and cannot distinguish between tensile and compressive meridians. The RHT constitutive model can characterize the entire deformation-damage-failure process of rock specimens, so its simulation results better reflect the true failure effects of rocks.

#### Mechanical properties of rock specimens

**Figure 10:**
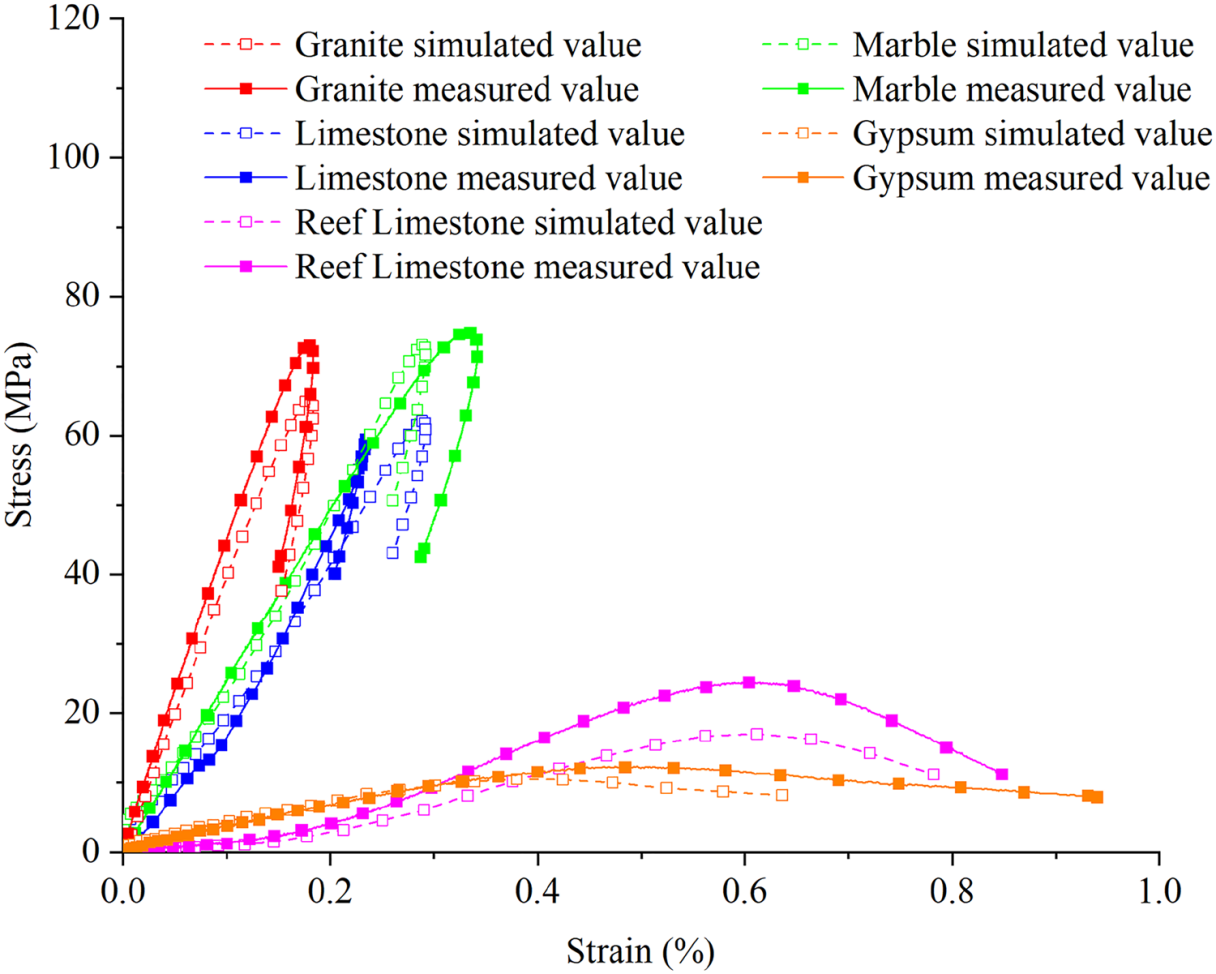
Stress–strain curves of simulated and measured value under the same impact pressure.

Through numerical simulation, stress–strain curves under 0.5 MPa impact pressure were obtained for samples of Shuangjiangkou rock, granite, marble, limestone, gypsum, and reef limestone, as shown in Fig. 10. There are subtle differences between the stress–strain curves in numerical simulation and those observed in experiments. The main reason for this is the presence of a long compaction stage in laboratory experiments, while in numerical simulations, the stress–strain curves start directly from the elastic phase, providing a more continuous representation of the deformation and failure process.

Combining the numerical simulation in Fig. 9 with the experimental rock sample failure process and damage cloud map, it is observed that the damage results from numerical simulation exhibit similarity with the laboratory test results in terms of strength characteristics and failure modes. This also indicates the accuracy of the numerical simulation, and the proposed constitutive model can be further used for numerical simulation experiments to study other dynamic properties of different rock samples.

**Figure 11:**
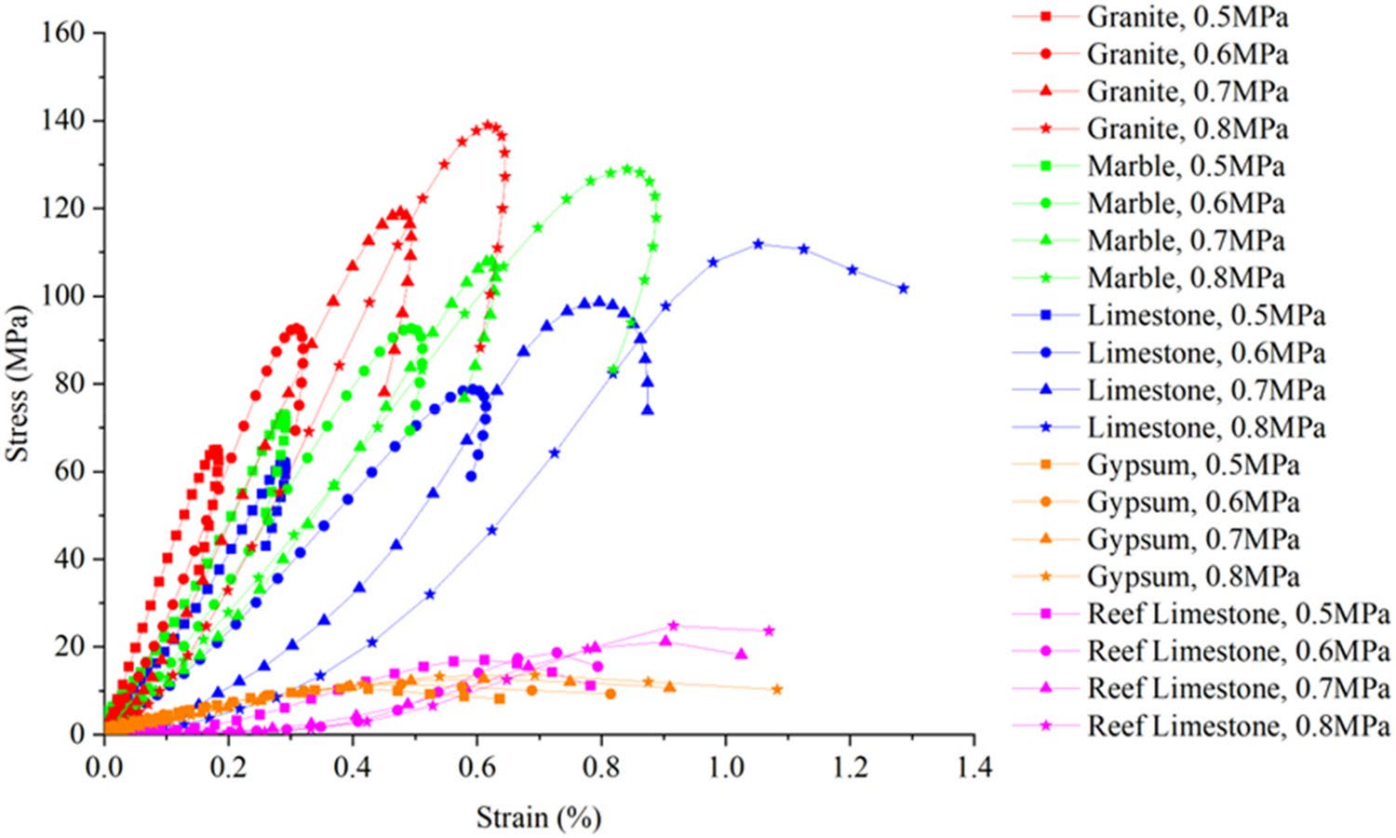
Stress–strain curves of different rock specimens in numerical simulation.

The stress–strain curves of five types of rock specimens calculated based on the RHT constitutive model are shown in Fig. 11. As the impact loading increases, the peak value of the dynamic stress–strain curve of rock specimens is shown to gradually increase. The stress–strain curves in the numerical simulation differ slightly from those in the experiment, mainly because the numerical simulation represents an idealized SHPB test, and its stress–strain curves start directly from the elastic stage, with a more continuous deformation and failure process.

Comparing the stress–strain curves of different rock specimens under the same impact gas pressure, it can be seen that as the strength of various rocks decreases, the peak value of the stress–strain curve gradually decreases, especially for gypsum and reef limestone, which will fail after only little deformation under the applied impact load. By comparing with the laboratory SHPB impact test, it can be found that when the impact gas pressure is 0.5 MPa and 0.6 MPa, the rock specimens have already undergone fragmentation failure, and when the impact gas pressure increases to 0.7 MPa and 0.8 MPa, some rock specimens are impacted to powder form.

The stress–strain curves of rock specimens in the numerical simulation results exhibit good similarity with the laboratory impact test data, so the RHT constitutive model parameters proposed in this paper can be used to study the other dynamic characteristics of rock specimens.

**Figure 12:**
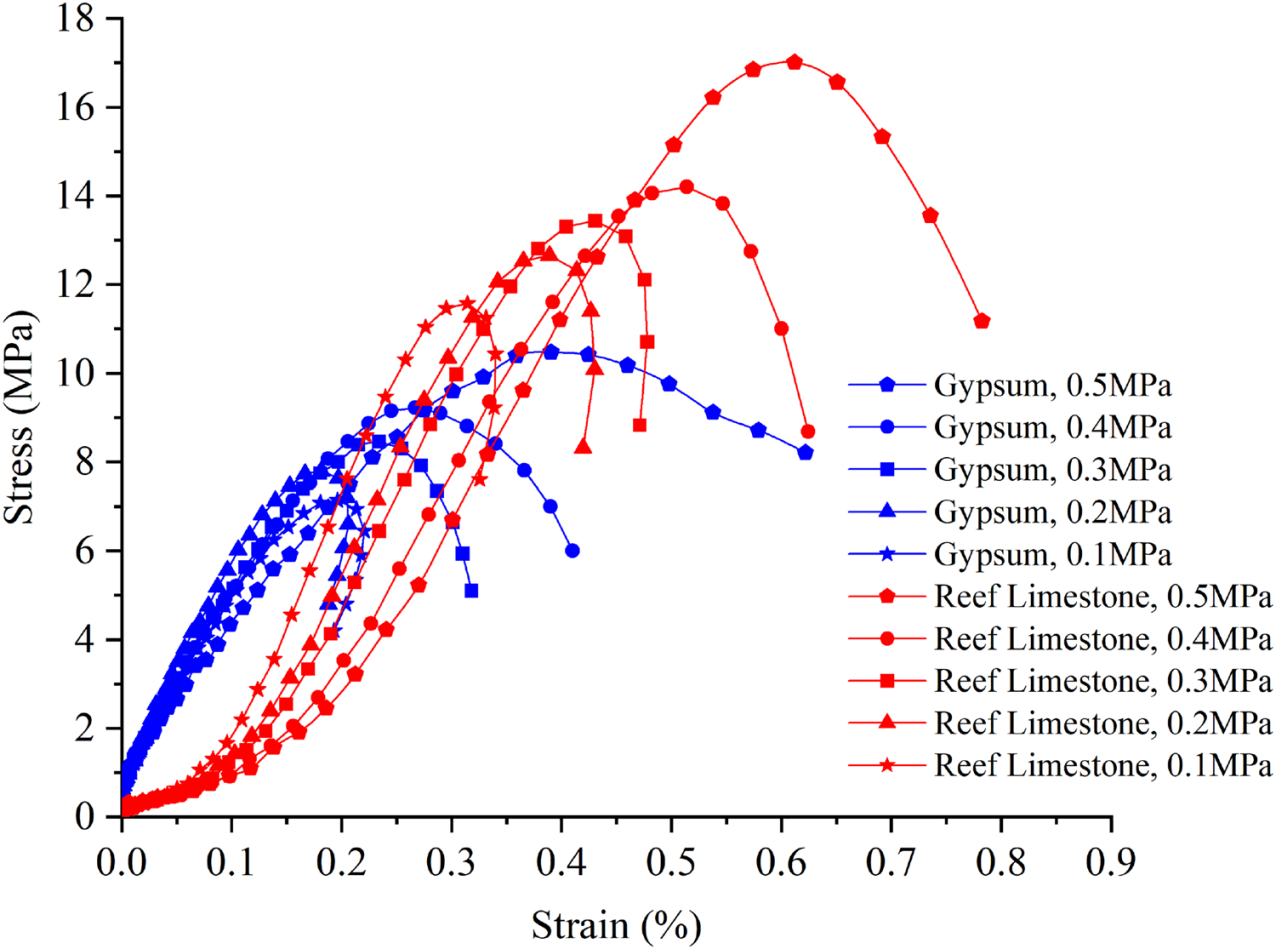
Numerical simulation stress–strain curves for gypsum and reef limestone.

The further to explore the critical impact pressure at which the stress–strain hysteresis loops of two types of soft rocks (gypsum and reef limestone) change from open to closed, the RHT constitutive model parameters of the aforementioned types of rock were selected. Numerical simulations were conducted at four impact pressures (0.4 MPa, 0.3 MPa, 0.2 MPa, and 0.1 MPa). The results are demonstrated in Fig. 12: as the impact loading decreases, the peak values and area enclosed by the hysteresis loops in the dynamic stress–strain curves of gypsum and reef limestone also gradually decrease. The stress–strain curve of reef limestone becomes a closed-loop at an impact loading of 0.3 MPa, indicating that the peak stress does not reach the dynamic yield strength of the material, and the material undergoes a rebound phenomenon. However, the hysteresis loop of the stress–strain curve for gypsum changes from open to closed at an impact load of 0.2 MPa.

## Conclusion

This paper investigates the mechanical properties and failure modes of different rock samples under the same static and dynamic loads through static mechanical parameter testing and SHPB dynamic impact tests. The accuracy of the indoor experimental results is verified through numerical simulations. The main conclusions obtained are as follows:

1. The impact resistance of the rock specimens is (in descending order): granite, marble, limestone, reef limestone, then gypsum. With the increase of impact pressure, the peak stresses of granite, marble, and limestone also increase. Under an impact load of 0.5–0.8 MPa, both reef limestone and gypsum turned into pulverized or powdered form, and the peak stresses at different strain rates are relatively low, without showing any significant regularity;
2. Both the HJC and RHT constitutive models can well simulate the laboratory impact test results of granite, marble, and limestone reasonably. However, gypsum and reef limestone are prone to failure under the HJC constitutive model, which cannot represent the deformation process of rock specimens. Nevertheless, the RHT constitutive model can accurately characterize the entire deformation and failure process of rock, making it better suited for reflecting the mechanical deterioration of rock under dynamic impact;
3. Numerical calculations were used to investigate the dynamic response characteristics of ordinary gypsum and reef limestone under impact loading. The stress–strain curve of reef limestone becomes a closed-loop curve when the impact load is reduced to 0.3 MPa, however, the hysteresis loop of the stress–strain curve for gypsum changes from open to closed at an impact load of 0.2 MPa.

## Data Availability

The datasets generated during and analyzed during the current study are available from the corresponding author on reasonable request.
